# Developmental outcomes after soybean oil vs mixed‐oil intravenous lipid emulsions in neonates: A secondary analysis of a clinical trial

**DOI:** 10.1002/jpen.70032

**Published:** 2025-11-10

**Authors:** Katie A. Huff, Charles Vanderpool

**Affiliations:** ^1^ Department of Pediatrics Indiana University School of Medicine Indianapolis Indiana USA; ^2^ Department of Pediatrics University of Cincinnati College of Medicine Cincinnati Ohio USA; ^3^ Division of Neonatology Cincinnati Children's Hospital Medical Center Cincinnati Ohio USA

**Keywords:** fat emulsions, infant, intestinal failure, intravenous, neurodevelopmental disorders, newborn

## Abstract

**Background:**

Fatty acids make up a significant portion of brain mass. The choice of lipid injectable emulsion alters a patient's fatty acid profile. In neonates with intestinal failure dependent on parenteral nutrition, this is particularly concerning given their rapid brain development.

**Methods:**

We randomly assigned 24 infants to receive soy oil lipid emulsion at 1 g/kg/day or mixed‐oil lipid emulsion containing soy, medium‐chain triglyceride, olive, and fish oils at 3 g/kg/day. We have previously reported data on intestinal failure–associated liver disease incidence with a 39‐day median follow‐up. We now report 3‐year follow‐up, nonprespecified neurodevelopmental screening outcomes using the Ages & Stages Questionnaires‐3, a test composed of five domains.

**Results:**

For this follow‐up analysis, 20 patients were included, 10 in each group. There was no difference in patient parameters between the groups at birth or screening. The mean ± SD peak direct bilirubin was not different between the two groups (soy 1.8 ± 1.4 mg/dl and mixed 1.2 ± 0.8 mg/dl; *P* = 0.265). There was also no difference when comparing the mean scores or rate of developmental delay for all five Ages & Stages Questionnaire‐3 domains between treatment groups. There was no correlation between peak direct bilirubin, gestational age, or days of lipid and domain scores.

**Conclusion:**

In this follow‐up study of children who had previously received soy oil lipid reduction or a mixed lipid, there was no difference in development at 3 years. Given that this is a secondary outcome from a pilot trial, additional studies powered for developmental differences are warranted.

## INTRODUCTION

Parenteral nutrition (PN) therapy is necessary to support life in the setting of intestinal failure (IF). The use of PN is associated with the development of IF‐associated liver disease (IFALD). Although the development of IFALD is multifactorial, intravenous lipid emulsions (ILEs) are noted to be a significant contributor to its development.^1^ Soybean oil–based ILE (SO‐ILE) is thought to contribute to IFALD given its higher ω‐6 polyunsaturated fatty acid (PUFA) content with less ω‐3 PUFA, elevated phytosterol levels, and lower levels of antioxidants including alpha tocopherol.^2^ Strategies for IFALD prevention include SO‐ILE reduction therapy and the use of alternate ILEs that contain less SO.^3^, ^4^, ^5^, ^6^ How the use of these alternate lipid‐dosing strategies affect neonatal outcomes, particularly neurodevelopmental outcomes, remains unknown.

In the pediatric patient, PUFAs contribute up to almost half of brain fatty acid content by age 5 years with fat making up 60% of overall brain mass.^7^, ^8^ Approximately half of all PUFAs in the brain are arachidonic acid (ARA) and docosahexaenoic acid (DHA).^7^ In neonates ARA and DHA are considered conditionally essential as the enzymes converting upstream fatty acids to these products have decreased activity.^9^ Different ILE products have variable DHA and ARA content.^10^ The ILE chosen, and the impact of ILE strategies used to reduce IFALD risk, is important to consider, as the patient fatty acid profile changes over time reflecting the ILE fatty acid content.^10^ Because of their rapid rate of brain growth and ongoing development, it is important to understand the influence of these ILE provision practice patterns on neonatal neurodevelopment. There is limited evidence comparing neurodevelopmental outcomes in a prospective manner in infants treated with different ILE provision strategies to reduce IFALD risk.

In our previous randomized pilot study we randomized neonates with intestinal pathology to receive either SO‐ILE reduction or an alternate ILE containing soy, medium‐chain triglyceride, olive, and fish oils (SO,MCT,OO,FO‐ILE) with the primary outcome of IFALD diagnosis.^5^ In this pilot study we found no difference in IFALD development but noted a decreased rate of rise in direct bilirubin levels over time in those patients treated with SO,MCT,OO,FO‐ILE.^5^ Importantly, we also noted decreased ARA and increased DHA levels in those treated with SO,MCT,OO,FO‐ILE compared with an SO‐ILE reduction.^5^

Although our center has adopted more widespread use of SO,MCT,OO,FO‐ILE in patients at risk for IFALD, the long‐term neurodevelopmental impact of these fatty acid alterations still remains unknown. The aim of this study is to report the post hoc follow‐up of our previous patient population by conducting neurodevelopmental screening at age 3 years comparing those patients who had received SO‐ILE at 1 g/kg/day or SO,MCT,OO,FO‐ILE at 3 g/kg/day as infants. We hypothesized that SO,MCT,OO,FO‐ILE is associated with decreased developmental delay in infants compared with SO‐ILE reduction.

## PATIENTS AND METHODS

### Trial design, treatment, and end points

We performed a secondary analysis of 24 patients who were randomized to receive SO‐ILE at 1 g/kg/day or SO,MCT,OO,FO‐ILE at 3 g/kg/day at the Riley Hospital for Children at Indiana University Health from 2018 to 2021. The full methods for our initial study have been previously described.^5^ This was a nonblinded, randomized pilot study investigating the influence of alternate lipid dosing on the development of the primary outcome of IFALD. Infants admitted to the level IV referral neonatal intensive care unit (NICU) at our institution who were estimated to need PN with lipid emulsion for at least 4 weeks were eligible. Infants were eligible if they had an anatomic intestinal diagnosis including intestinal atresia, gastroschisis, omphalocele, volvulus, or an ischemic/perforation diagnosis including necrotizing enterocolitis or spontaneous intestinal perforation. In addition to diagnosis, infants were only eligible if they required an abdominal procedure that involved the intestines or abdominal wall. Infants were ineligible if they had high risk for mortality, cholestasis development, or liver disease at baseline, including weight <750 g, culture‐proven sepsis, renal failure requiring dialysis, prostaglandin‐dependent heart disease, aspartate aminotransferase or alanine aminotransferase greater than five times the upper limit of normal, a direct bilirubin level >2 mg/dl, international normalized ratio >2.0, or triglyceride level >250 mg/dl.

Enrolled infants were randomized to receive one of two lipid therapies. Our exposure of interest was mixed‐oil ILE containing fish oil (SO,MCT,OO,FO‐ILE) at 3 g/kg/day. Our comparator was SO‐ILE at 1 g/kg/day. The study ILE was continued for a maximum of 12 weeks or until one of the following occurred: PN was discontinued, direct bilirubin level rose to ≥3 mg/dl on two occasions, or they were discharge from our facility. The remainder of PN and care was at the discretion of the NICU team. Block randomization with varying block sizes of two and four was used with infants stratified based on diagnosis group (anatomic vs ischemic/perforation). Premade, sealed envelopes were used for randomization.

### Primary and secondary outcomes

In addition to the primary outcome of IFALD, and numerous secondary outcomes previously reported,^5^ an additional predefined secondary outcome was neurodevelopmental differences between treatment groups at age 2 years. Our primary outcome for this study was developmental delay determined at age 3 years defined by the Ages and Stages Questionnaires, third edition (ASQ‐3) with the threshold below the cutoff for need for referral.^11^ Our primary outcome differed from our prespecified secondary outcome of ASQ‐3 screening at age 2 years because of a delay in obtaining the initial screening with the protocol amended and age updated to 3 years of chronological age to promote consistency among patients.

The specific ASQ‐3 questionnaire used was determined based on patient age on the day of screening as outlined by the questionnaire instructions. Questionnaires were completed using parent or guardian responses over the phone as obtained by research personnel. In addition, completed ASQ‐3 forms entered into the electronic medical record were used for study purposes if they were obtained within the study age window by another medical professional. The ASQ‐3 assesses five developmental domains including communication, gross motor, fine motor, problem solving, and personal social.^11^ Each domain section consists of 10 questions with response options of “yes,” “not yet,” or “sometimes” with regard to a child's ability to perform the specified task. For score calculation, “yes” answers are awarded 10 points, “sometimes” 5 points, and “not yet” 0 points for a possible total score range of 0–60 for each domain. ASQ‐3 instructions were followed to calculate scores with adjustments made if one to two responses per domain section were missing or unanswered. Per instructions, if more than two responses were blank the section was not scored.^12^ Scores were interpreted based on the ASQ‐3 summary for score threshold to qualify for one of three categories: above the cutoff or developmentally appropriate, close to the cutoff or monitoring zone, and below the cutoff with need for referral or termed as developmental delay within this study. Parents were notified of their child's score and interpretation immediately at questionnaire completion. If the patient scored in the monitoring or referral zones the parent was instructed to follow‐up with their pediatrician and report a concern for developmental delay if no delay was previously known or diagnosed.

Additional secondary outcomes reported as part of this study include the rate of overall development delays, prior diagnosis of developmental delay, and the rate of therapies received. The rate of these outcomes was based on parental report. To compare the two populations in this study, we report multiple parameters reported in our previous study including patient growth, hospital complications, and nutrition parameters. All of these variables were only from the initial patient hospitalization in infancy when they were enrolled in the original randomized study. Patient growth was noted as weight, length, and head circumference (occipitofrontal circumference) velocity calculated from enrollment until the end of the study period divided by the number of days of study ILE. Hospital complications reported include intestinal length resected, number of intestinal surgeries, culture‐proven bacteremia, and postnatal cytomegalovirus diagnosis. Nutrition parameters include the total days of any ILE, days of study ILE, total days of PN, need for PN >60 days, average delivered energy (enteral + parenteral), and average ILE dose received. Essential fatty acid deficiency was also reported as defined in our original study of a triene:tetraene ratio >0.05.

### Statistical analysis, sample size, and oversight

**Figure 1:**
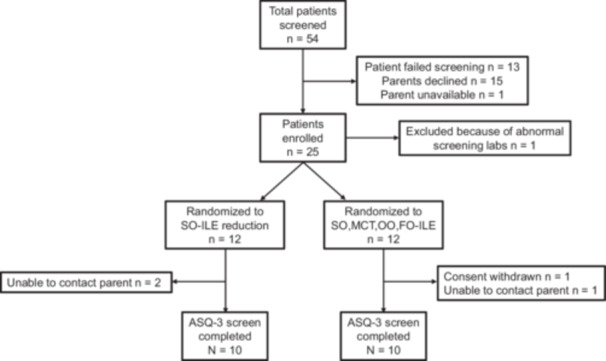
Schematic of patient enrollment, randomization, and follow‐up. ASQ‐3, Ages and Stages Questionnaire, third edition; SO‐ILE, soybean oil–based intravenous lipid emulsion; SO,MCT,OO,FO‐ILE, soy, medium‐chain triglyceride, olive, and fish oils ILE.

This study was approved by the Indiana University Institutional Review Board and received Investigational New Drug application approval through the US Food and Drug Administration (FDA). This study was also registered with ClinicalTrials.gov (NCT03387579). All data were analyzed using GraphPad Prism version 10.4.1 (GraphPad Software; graphpad.com). All continuous variables are presented as mean ± SD and assessed using *t* test or Mann‐Whitney *U* nonparametric analysis dependent on the normality of the data distribution. Bivariate variables are expressed as numbers and percentages and were analyzed using the Fisher exact test. A *P* value of 0.05 was used to define significance. The Spearman correlation was used to assess the association between ASQ‐3 domain scores and peak direct bilirubin, birth gestational age, birth weight, days of study lipid therapy, and average ILE dose. These parameters were chosen based on their known relation to developmental delay risk (birth weight and gestational age) or their relation to our original randomized trial (peak direct bilirubin, days of study ILE, and average ILE dose). The original randomized 1:1 pilot trial had a sample size goal of 12 patients per ILE group.^5^, ^13^ The current study analyzes patients from the original randomized pilot trial available for follow‐up. A total of 25 infants were enrolled in the original study with 24 being randomized 1:1 to ILE groups.^5^ Patients were treated with the assigned study lipid between December 2018 and February 2021. Two infants in each ILE group were lost to follow‐up, leaving 10 infants in each ILE group for ASQ‐3 screening (Figure 1). ASQ‐3 screening occurred between November 2021 and March 2024.

## RESULTS

Patients in each group had a similar chronological age at ASQ‐3 screening (Table 1). The screened patients were similar with regard to gestational age and birth weight. The gestational age was also similar between the groups when including those infants who were not screened (SO‐ILE 35.1 ± 3.5 weeks and SO,MCT,OO,FO‐ILE 33.5 ± 5.2 weeks; *P* = 0.213). When comparing diagnosis frequency between those who were screened (Table 1) and the entire cohort (*P* = 0.190) there was also no significant difference with regard to diagnosis between the treatment groups.

**Table 1:** Demographic and patient data from time in original randomized study.

Parameter	SO,MCT,OO,FO‐ILE (*n* = 10)	SO‐ILE (*n* = 10)	*P* value
Birth gestational age, median (IQR), weeks	34.7 (29.8–37.4)	36.7 (34.6–37.1)	0.21^a^
Birth weight, median (IQR), g	2340.0 (1192.5–2970.0)	2735.0 (2025.3–2972.5)	0.95^b^
Growth velocity during study, median (IQR)			
Weight, g/day	25.5 (21.0–38.3)	25.1 (9.6–28.0)	0.21^b^
Length, cm/day	0.14 (0.06–0.18)	0.14 (0.06–0.14)	0.45^a^
OFC, cm/day	0.08 (0.07–0.11)	0.1 (0.02–0.11)	1.00^a^
Peak direct bilirubin in NICU, mg/dl	1.2 (0.4–1.5)	1.3 (0.7–3.1)	0.26^b^
Diagnosis, *n* (%)			
Atresia	4 (40.0)	2 (20.0)	0.09^c^
Gastroschisis	2 (20.0)	6 (60.0)	
NEC	2 (20.0)	0 (0)	
Omphalocele	1 (10.0)	0 (0)	
Perforation	0 (0)	2 (20.0)	
Volvulus	1 (10.0)	0 (0)	
Hospital course complications			
Intestinal length resected, median (IQR), cm	9.0 (0–26.3)	0 (0–12.5)	0.22^c^
Number of intestinal surgeries, median (IQR)	2.0 (1.0–3.0)	1.0 (1.0–1.3)	0.12^a^
Culture‐proven bacteremia, *n* (%)	1 (10)	0 (0)	1.00^c^
Postnatal CMV diagnosis, *n* (%)	0 (0)	1 (10)	1.00^c^
Nutrition parameters			
Total days of ILE, median (IQR)	26.5 (18.3–70.8)	21.0 (12.0–33.5)	0.38^a^
Days of study ILE, median (IQR)	22.0 (13.3–66.3)	18.0 (9.3–27.3)	0.36^a^
Total days of PN, median (IQR)	24.5 (18.3–68.5)	21.0 (12.0–30.5)	0.45^a^
Need for PN >60 days, *n* (%)	4 (40)	1 (10)	0.30^c^
Average delivered energy, median (IQR), kcal/kg/day	114.0 (109.3–118.0)	97.5 (92.0–108.3)	0.02^b^
Average ILE dose, g/kg/day	2.6 (2.5–2.8)	1.0 (1.0–1.1)	<0.001^a^
Essential fatty acid deficiency *n*/*N* (%)	0/9 (0)	1/7 (14)	0.47^c^

Abbreviations: CMV, cytomegalovirus; ILE, intravenous lipid emulsion; NEC, necrotizing enterocolitis; NICU, neonatal intensive care unit; OFC, occipitofrontal circumference; PN, parenteral nutrition; SO‐ILE, soybean oil–based ILE; SO,MCT,OO,FO‐ILE, soy, medium‐chain triglyceride, olive, and fish oils ILE.

^a^ *P* values determined by Mann‐Whitney test.

^b^ *P* values determined by *I* test.

^c^ *P* values determined by Fisher exact test.

**Figure 2:**
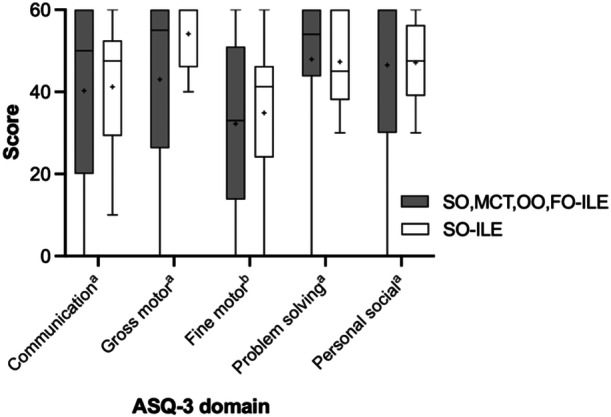
ASQ‐3 score distribution comparing lipid groups. Box and whiskers plot of ASQ‐3 scores with boxes representing the 25th and 75th percentiles, lines representing the median, and whiskers representing the minimum and maximum scores. Pluses note the mean. All *P* values >0.05. ^a^ *P* values determined by Mann‐Whitney test. ^b^ *P* values determined by *t* test. ASQ‐3, Ages and Stages Questionnaire, third edition; SO‐ILE, soybean oil–based intravenous lipid emulsion; SO,MCT,OO,FO‐ILE, soy, medium‐chain triglyceride, olive, and fish oils ILE.

The distribution of ASQ‐3 scores is depicted in Figure 2. Mean ± SD communication scores were 40.3 ± 22.4 for SO,MCT,OO,FO‐ILE and 41.2 ± 17.7 for SO‐ILE (*P* = 0.720). Mean gross motor scores were SO,MCT,OO,FO‐ILE 43.0 ± 24.2 and SO‐ILE 54.1 ± 8.19 (*P* = 0.500). Mean fine motor scores were SO,MCT,OO,FO‐ILE 32.2 ± 21.6 and SO‐ILE 34.9 ± 19.1 (*P* = 0.774). Mean problem solving scores were SO,MCT,OO,FO‐ILE 47.9 ± 19.5 and SO‐ILE 47.3 ± 11.1 (*P* = 0.457). Mean personal social scores were SO,MCT,OO,FO‐ILE 46.5 ± 20.6 and SO‐ILE 47.1 ± 10.5 (*P* = 0.475). A similar number of infants had known developmental delays at the time of ASQ‐3 screen and were actively receiving therapies (Table 2). There was no difference in the rate of developmental delay or scores below the ASQ‐3 cutoff in any domain when comparing the treatment groups (Table 2). Table 3 notes the correlation between patient parameters and ASQ‐3 results. There was no significant correlation between peak direct bilirubin, birth gestational age, birth weight, days of study lipid therapy, or average ILE dose and any of the five ASQ‐3 domains.

**Table 2:** Patient parameters at time of ASQ‐3 and rates of developmental delay by domain for each treatment group.

Parameter	SO,MCT,OO,FO‐ILE (*n* = 10)	SO‐ILE (*n* = 10)	*P* value
Median age at ASQ‐3, median (IQR), months	43.5 (38.4–48.7)	38.5 (36.0–43.6)	0.11^a^
Receiving therapies at time of ASQ‐3, *n* (%)	5 (50.0)	4 (40.0)	
ABA, *n*	1	0	1.00^b^
Occupational, *n*	3	3	
Physical, *n*	4	2	
Speech, *n*	3	4	
Known developmental delays or diagnoses affecting ASQ‐3, *n* (%)	5 (50.0)	4 (40.0)	1.00^b^
Arthrogryposis, *n*	1	0	
Autism, *n*	1	1	
Tracheostomy, *n*	0	1	
Trisomy 21, *n*	1	0	
Rate of developmental delay on ASQ‐3, *n*/*N* (%)			
Communication	4/10 (40.0)	2/10 (20.0)	0.63^b^
Gross motor	3/10 (30.0)	0/9 (0)	0.21^b^
Fine motor	4/10 (40.0)	2/10 (20.0)	0.63^b^
Problem solving	1/9 (11.1)	1/9 (11.1)	1.00^b^
Personal social	3/10 (30.0)	1/10 (10.0)	0.58^b^
Any developmental delay, *n*/*N* (%)	6/10 (60.0)	3/10 (30.0)	0.37^b^

Abbreviations: ABA, applied behavioral analysis; ASQ‐3, Ages and Stages Questionnaire, third edition; SO‐ILE, soybean oil–based intravenous lipid emulsion; SO,MCT,OO,FO‐ILE, soy, medium‐chain triglyceride, olive, and fish oils ILE.

^a^ *P* values determined by *t* test.

^b^ *P* values determined by Fisher exact test.

**Table 3:** Correlation of patient characteristics and lipid treatment with ASQ‐3 results.

Parameter	ASQ‐3 domain	Spearman correlation coefficient	*P* value
Peak direct bilirubin	Communication	0.32	0.18
	Gross motor	0.06	0.81
	Fine motor	−0.13	0.58
	Problem solving	−0.04	0.88
	Personal social	0.13	0.58
Birth gestational age	Communication	0.08	0.73
	Gross motor	0.38	0.11
	Fine motor	0.38	0.10
	Problem solving	0.19	0.45
	Personal social	−0.03	0.92
Birth weight	Communication	0.09	0.71
	Gross motor	0.35	0.14
	Fine motor	0.33	0.15
	Problem solving	0.30	0.22
	Personal social	0.05	0.83
Days of study lipid therapy	Communication	0.12	0.61
	Gross motor	0.16	0.53
	Fine motor	−0.05	0.85
	Problem solving	0.06	0.82
	Personal social	0.03	0.89
Average ILE dose	Communication	0.02	0.92
	Gross motor	−0.19	0.45
	Fine motor	−0.07	0.79
	Problem solving	0.13	0.60
	Personal social	0.01	0.97

*Note*: Data analyzed using the Spearman correlation and presented as the Spearman correlation coefficient.

Abbreviations: ASQ‐3, Ages and Stages Questionnaire, third edition; ILE, intravenous lipid emulsion.

## DISCUSSION

We report here the neurodevelopmental outcomes of neonatal patients treated with SO‐ILE reduction or SO,MCT,OO,FO‐ILE therapies using the ASQ‐3. This study is a secondary analysis of our prior randomized pilot trial assessing the diagnosis of IFALD in this high‐risk patient population. We noted no baseline differences between the two groups with regard to diagnosis, peak direct bilirubin, or days of lipid therapy. This is important to note as developmental outcomes have been tied to intestinal diagnoses, with one meta‐analysis showing increased rates of adverse developmental outcomes in the setting of IF and surgical necrotizing enterocolitis in particular.^14^ Although we did not find a correlation with regard to peak direct bilirubin level and ASQ‐3 results in any domain, prior studies have noted increased rates of developmental delays in extremely low birthweight infants treated with SO‐ILE or SO,MCT,OO,FO‐ILE who developed elevated direct bilirubin levels.^15^

Additionally, there was no baseline difference with regard to gestational age or birthweight in our cohort. We also did not find a correlation with regard to developmental delays and gestational age or birthweight. This is different than would be expected, as it is well established that the rate of developmental delay increases with decreasing birthweight and birth gestational age.^16^ This could, in part, be because of the overall more moderate or late preterm mean gestational ages of the cohorts. It should be noted, however, that even moderate to late preterm infants are at increased risk of developmental delays in childhood when compared with term infants.^17^ Additionally, we chose to exclude those infants with a birth weight <750 g given their increased risk for IFALD development, eliminating a group of infants at high risk for negative developmental outcomes.^18^

Previous groups have also reported neurodevelopmental outcomes of children who had received either SO‐ILE or SO,MCT,OO,FO‐ILE as infants. Thanhaeuser et al reported developmental outcomes at 12 months, 24 months, and preschool age in previous extremely low birthweight infants who had received either SO‐ILE or SO,MCT,OO,FO‐ILE.^19^, ^20^ When comparing treatment groups over time, they noted no difference in developmental outcomes at any time point using the Bayley Scales of Infant and Toddler Development III (Bayley) and the Kaufman Assessment Battery for Children II.^19^, ^20^ Other groups have also compared premature infants who received SO‐ILE or SO,MCT,OO,FO‐ILE with no differences in neurodevelopmental outcomes noted.^21^ One group did note a decreased odds of death or any neurodevelopmental impairment in a pre/post cohort in those receiving SO,MCT,OO,FO‐ILE compared with SO‐ILE. However, this was a retrospective cohort nonrandomized study and has not been replicated in randomized studies as noted.^22^

It is important to consider that in our study, unlike the studies mentioned above, the SO‐ILE group received reduced doses at a goal of 1 g/kg/day. Blackmer et al. reported ASQ‐3 findings at ages 2–5 years in children who previously received SO‐ILE at reduced doses. They noted 5.6%–17.7% of patients were below the cut off for a given ASQ‐3 domain.^23^ In our cohort we noted a wider range of delays in the various domains of 0%–40% with no difference noted when comparing between the two treatment groups. Other groups have compared neurodevelopmental outcomes after SO‐ILE reduction or standard SO‐ILE dosing at 3 g/kg/day, again finding no difference in neurodevelopmental outcomes.^24^

To assess developmental outcomes in our study we used the ASQ‐3, a screening tool. Although a developmental assessment such as the Bayley is considered the gold standard, it can be time consuming and expensive to conduct.^25^, ^26^ Studies have shown mixed results with regard to the reliability of the ASQ compared with the Bayley. In a meta‐analysis including 43 studies comparing the ASQ with various developmental screens, including the Bayley, the ASQ was noted to have sensitivity and specificity to predict developmental delays that aligns with the American Academy of Pediatrics (AAP) goal of 70%–80%.^26^, ^27^ This shows the ASQ‐3 is an acceptable screening tool to consider.

Although the ASQ‐3 is an acceptable screening tool per AAP standards, it is less sensitive and specific than a developmental screen. Given this difference, when assessing neurodevelopmental outcomes a larger patient population may be needed when the ASQ‐3 is used in comparison with a developmental assessment tool such as the Bayley.^28^ As data were analyzed from a pilot randomized trial, our study is potentially underpowered because of a small sample size that increases the risk of failing to detect a true difference between ILE groups. It is worth noting that although there is no statistically significant difference in developmental delays between our groups, there is an overall nonsignificant increase in the rate of developmental delay in those patients receiving SO,MCT,OO,FO‐ILE (60% vs SO‐ILE 30%). Although this could be attributed to the nonsignificant younger gestational age and lower birthweight in this group, the small sample size of this study limits our ability to fully understand this finding. Additionally, the analyses in this study were univariate, which would not account for confounders that may influence our findings. Future studies should consider a larger sample size if planning to assess developmental outcomes. Additional limitations of our study include the inclusion of infants with known conditions that could affect future development, including trisomy 21 and arthrogryposis. Given that the primary outcome was IFALD development in our pilot study, known conditions increasing the risk for developmental delays were not a reason for exclusion. This should again be a consideration in a study with a larger patient population.

## CONCLUSION

In this follow‐up study of children who had previously received either SO lipid reduction or a mixed‐oil lipid emulsion containing fish oil at standard dosing, we did not find a statistically significant difference between ILE groups in developmental outcomes at age 3 years using the ASQ‐3. Given the increasing use of alternate lipid emulsion dosing, strong consideration should be given to conducting a future study powered for developmental differences in these complex neonates.

## AUTHOR CONTRIBUTIONS

Katie A. Huff contributed to the conceptualization, investigation, funding acquisition, methodology, original draft, validation, review and editing, formal analysis, data curation, visualization, and project administration. Charles Vanderpool contributed to the review and editing, conceptualization, investigation, methodology, project administration, and supervision.

## CONFLICT OF INTEREST STATEMENT

Katie A. Huff was a previous advisor for Baxter. Charles Vanderpool declares no conflicts of interest.
